# YouTube videos as a source of medical information during the Ebola hemorrhagic fever epidemic

**DOI:** 10.1186/s40064-015-1251-9

**Published:** 2015-08-28

**Authors:** Sajan Jiv Singh Nagpal, Ahmadreza Karimianpour, Dhruvika Mukhija, Diwakar Mohan, Andrei Brateanu

**Affiliations:** Department of Internal Medicine, Cleveland Clinic Foundation, 9500 Euclid Ave, NA-10, Cleveland, OH 44195 USA; Bloomberg School of Public Health, Johns Hopkins University, Baltimore, MD USA

**Keywords:** Patient education, Ebola, Epidemics, Social media, Multimedia, Ebola hemorrhagic fever

## Abstract

The content and quality of medical information available on video sharing websites such as YouTube is not known. We analyzed the source and quality of medical information about Ebola hemorrhagic fever (EHF) disseminated on YouTube and the video characteristics that influence viewer behavior. An inquiry for the search term ‘Ebola’ was made on YouTube. The first 100 results were arranged in decreasing order of “relevance” using the default YouTube algorithm. Videos 1–50 and 51–100 were allocated to a high relevance (HR), and a low relevance (LR) video group, respectively. Multivariable logistic regression models were used to assess the predictors of a video being included in the HR vs. LR groups. Fourteen videos were excluded because they were parodies, songs or stand-up comedies (n = 11), not in English (n = 2) or a remaining part of a previous video (n = 1). Two scales, the video information and quality and index and the medical information and content index (MICI) assessed the overall quality, and the medical content of the videos, respectively. There were no videos from hospitals or academic medical centers. Videos in the HR group had a higher median number of views (186,705 vs. 43,796, p < 0.001), more ‘likes’ (1119 vs. 224, p < 0.001), channel subscriptions (208 vs. 32, p < 0.001), and ‘shares’ (519 vs. 98, p < 0.001). Multivariable logistic regression showed that only the ‘clinical symptoms’ component of the MICI scale was associated with a higher likelihood of a video being included in the HR vs. LR group.(OR 1.86, 95 % CI 1.06–3.28, p = 0.03). YouTube videos presenting clinical symptoms of infectious diseases during epidemics are more likely to be included in the HR group and influence viewers behavior.

## Background

Ebola virus, a member of the *Filoviridae* family is one of the most virulent pathogens in humans (Feldmann and Geisbert 2011). The ongoing Ebola hemorrhagic fever (EHF) epidemic that started in 2014 is the largest in history, with its epicenter being located primarily in West African countries (Nyenswah et al. 2014). The case fatality rate for the infection with Zaire species of the Ebola virus, which is the strain causing the current epidemic, has previously been reported to be 80–90 percent (Bray and Murphy 2007). The alarming mortality rates as well as the spread of the virus outside Africa has created much panic among the general public and healthcare institutions around the world, especially because the presenting signs and symptoms can mimic a variety of other conditions. The frenzy surrounding the spread of the Ebola virus has been compared to that seen in response to the emergence of the acquired immune deficiency syndrome in the 1980s (Gonsalves and Staley 2014). Widespread media coverage resulted in ‘Ebola’ becoming the third most searched word on the Internet (2015). Simultaneously, Ebola related topics have emerged as popular subjects on different video sharing websites such as YouTube, the third most popular website in the world (2015). Judging by its propensity to create panic and fear, it becomes all the more important for physicians and healthcare organizations to be aware of the content and quality of information available online and be able to create accurate and effective data sources to guide the general public. However, a very limited number of studies have assessed the usefulness of YouTube in the dissemination of medical information and these studies have been limited to chronic diseases such as rheumatoid arthritis, hypertension, renal stones, skin cancer and anorexia nervosa (Singh et al. 2012; Kumar et al. 2014; Sood et al. 2011; Murugiah et al. 2011; Basch et al. 2015; Syed-Abdul et al. 2013).

We analyzed the source and quality of medical information about Ebola hemorrhagic fever (EHF) disseminated on YouTube and the video characteristics that influence viewers’ behavior.

## Methods

The online video hosting resource YouTube (http://www.youtube.com) was accessed on December 9th, 2014 from Cleveland, OH, USA. A new account was made for the purpose of the study. An inquiry for the search term “Ebola” was requested, and the results were sorted in decreasing order of “relevance” using the default YouTube algorithm. Out of the approximately 1.35 million results, we selected the first 100 videos and saved them in a playlist because search results on YouTube can change from day-to-day. Uniform resource locators (URLs) of the top 100 videos were also saved separately for backup.

According to YouTube, watch time is an important metric for video promotion and sorting and the algorithm used for suggesting videos prioritizes videos that lead to a longer overall viewing session over those that receive more clicks. While viewers benefit from a more enjoyable content, creators benefit from a more focused, engaged audience (2015). As a consequence, our inquiry was stratified using the default search filters. It has been shown that the majority of web surfers do not look beyond the third page of search results, therefore the first 100 videos were considered to be representative of viewers’ behavior (Fox and Duggan 2015). Only videos in the English language were included. All videos were viewed and analyzed for content by two independent physicians (SJSN and AK) and the results were then consolidated. Discrepancies were resolved by arbitration in the presence of a third reviewer (DM). Videos were allocated to two groups for comparison, a high relevance (HR) (videos 1–50), and a low relevance (LR) group (videos 51–100), respectively.

### Video sources

The source of each video was identified. Previous studies have used ‘patient views’ and ‘university/hospital channels’ as sources of classifying the videos (Kumar et al. 2014; Sood et al. 2011; Murugiah et al. 2011). Since there were no available interviews with Ebola infected patients and no university/hospital channels in the videos included in our study, these categories were excluded. The remaining categories included ‘Independent Users or Websites’ (IU/W) (e.g. homemade videos, un-official news websites) and ‘Government Agencies/News Agencies’ (GA/NA) (e.g. Reuters, CNN, White House).

### Video assessment

Since there were no established scales to evaluate the quality of information on video sharing websites, authors devised two scales, the Video information and quality index (VIQI) and the medical information and content index (MICI) scale.

The VIQI scale assesses the overall quality of the video, and addresses each component of the Global Quality Scale (GQS) separately (Kumar et al. 2014). The GQS was used in previous studies to assess the quality of a video, but was actually designed to evaluate the information quality on websites. The VIQI scale uses a 5-point Likert scale from 1 (poor quality) to 5 (high quality) to assess the following video characteristics: flow of information, information accuracy, quality (one point each for use of still images, use of animation, interview with individuals in the community, video captions, and use of a report summary), and precision (level of coherence between video title and content).

The MICI scale uses a 5-point Likert scale from 1 (poor quality) to 5 (high quality) to assess the following components of the medical information included in the videos: prevalence, transmission, clinical symptoms, screening/testing, and treatment/outcomes of the Ebola virus infection.

### Data collection

Data was collected and managed using REDCap electronic data capture tools hosted at Cleveland Clinic. REDCap (Research Electronic Data Capture) is a secure, web-based application designed to support data capture for research studies, providing: (1) an intuitive interface for validated data entry; (2) audit trails for tracking data manipulation and export procedures; (3) automated export procedures for seamless data downloads to common statistical packages; and (4) procedures for importing data from external sources.

### Statistical analysis

For continuous variables, means with standard deviations or medians with interquartile ranges were calculated, as appropriate. For hypotheses testing, we calculated differences in means using the unpaired Student T test and the test of medians for non-parametric data. Variables were compared between the HR and LR groups. Multivariable logistic regression analysis was conducted to investigate the predictive power of each video characteristic on the YouTube video relevance.

The data analysis was done using stata 13.1 (StataCorp. 2013. *Stata Statistical Software: Release 13*. College Station, TX: StataCorp LP.). A p value <0.05 was considered significant.

## Results

Out of the 100 videos screened, 86 unique videos with a total duration of 25.96 h and 22,888,539 views were included. Fourteen videos were excluded because they were parodies, songs or stand-up comedies (N = 11), not in English language (N = 2) or a remaining part of a previous video (N = 1). The kappa coefficient of agreement between the two physicians that initially reviewed the videos was 0.82. Forty-two (84.0 %) and 44 (88.0 %) videos were included in the HR and LR group, respectively. Fifty videos (58.13 %) originated from IU/W and 36 (41.87 %) from GA/NA sources.

The videos characteristics are summarized in Table 1. Compared to the LR, the HR video group had a higher median number of views (186,705 vs. 43,796, p < 0.001), more ‘likes’ (1119 vs. 224, p < 0.001), ‘dislikes’ (105 vs. 25, p < 0.001), time watched (2 vs. 0.5 years, p < 0.01), channel subscriptions (208 vs. 32, p < 0.001) and ‘shares’ (519 vs. 98, p < 0.001).

**Table 1:** Descriptive analysis of the YouTube videos

Variables	High relevance videos N = 42 Median (IQR) or mean (SD)	Low relevance videos N = 44 Median (IQR) or mean (SD)	P value
Video source			
GA/NA	18	18	0.85
IU/W	24	26	
Video characteristics			
Number of views	186,705 (52,846–607,829)	43,796 (10,709–86,264)	<0.001
Number of channel subscription	517,489 (57,399–972,098)	118,650 (41,218–928,585)	0.06
Number of likes	1119 (373–2913)	224 (87–568)	<0.01
Number of dislikes	105 (43–348)	25 (8–53)	<0.01
Like/dislike ratio	8.2 (5.1–18.8)	10.06 (7.9–14.9)	0.74
Number of likes/1000 views	6.8 (2.9–9.5)	7.90 (3.8–12.8)	0.20
Number of dislikes/1000 views	0.6 (0.3–1.1)	0.6 (0.2–1.2)	0.74
Time watched (years)	2 (0.6–5.2)	0.5 (0.2–0.9)	<0.01
Average view duration (s)	323 (207–529)	392 (221–664)	0.34
Number of new subscriptions	208 (36–717)	32 (9–86)	<0.001
Number of shares	519 (128–1026)	98 (52–233)	<0.01
Video information and quality index (VIQI) content assessment			
Flow	3.67 (1.05)	3.11 (0.81)	0.01
Information	2.71 (1.58)	2.30 (1.05)	0.38
Quality	2.21 (1.00)	2.16 (0.91)	0.84
Precision	3.00 (1.51)	2.02 (0.85)	<0.01
Total score	11.60 (4.64)	9.59 (2.44)	0.16
Medical information and content index (MICI) content assessment			
Prevalence	1.93 (1.88)	1.16 (1.64)	0.03
Transmission	2.12 (1.90)	1.25 (1.70)	0.04
Clinical symptoms	1.88 (1.98)	0.68 (1.43)	<0.01
Screening/testing	0.62 (1.12)	0.341 (0.89)	0.12
Treatment/outcomes	1.40 (1.73)	0.75 (1.40)	0.03
Total score	7.95 (7.80)	4.18 (5.69)	0.03

*GA/NA* Government/News agencies, *IU/W* Individual users/Websites

Videos in the HR group scored significantly higher in the ‘flow’ (3.67 ± 1.05 vs. 3.11 ± 0.81, p = 0.01) and ‘precision’ (3.00 ± 1.51 vs. 2.02 ± 0.85, p < 0.01) components of the VIQI score but there was no difference in the total VIQI score between the HR and LR groups. The MICI score was higher in the HR group when compared to the LR group (7.95 ± 7.80 vs. 4.18 ± 5.69, p = 0.03).

Of the seven video characteristics included in the backward multivariable logistic regression analysis, only the ‘clinical symptoms’ score (Table 2) was associated with a higher likelihood for a video to be included in the HR group (OR 1.86, 95 % CI 1.06–3.28, P = 0.03).

**Table 2:** Factors associated with high relevance YouTube videos

Variables	Odds ratio (95 % CI)	P value
Video source	1.46 (0.49–4.33)	0.49
Video information and quality index (VIQI) total score	1.73 (0.68–4.42)	0.25
Medical information and content index (MICI) scores		
Prevalence	0.90 (0.58–1.39)	0.62
Transmission	0.89 (0.56–1.38)	0.57
Clinical symptoms	1.86 (1.06–3.28)	0.03
Screening/testing	0.83 (0.42–1.65)	0.60
Treatment/outcomes	0.82 (0.46–1.48)	0.52

Multivariable logistic regression analysis

## Discussion

Our study provides a detailed analysis of the YouTube videos as a source of medical information. As the use of electronic multimedia infiltrates every aspect of medicine and healthcare, it is essential to study the accuracy and quality of the information delivered. During the 2014 EHF epidemic, Internet was considered to be the best tool available to health experts to predict a disease outbreak (2015). However, penetration of the Internet and social media is extremely poor in West Africa, which unfortunately was also the heart of the EHF epidemic. Improvement in the Internet connectivity and communication amongst healthcare agencies was identified by the WHO as being key in the fight against EHF (2015). While Rodriguez-Morales et al. (2014) looked at the behavior of the Internet users searching for EHF on social media websites such as Twitter, Oyeyemi et al. (2014) expressed concern about the amount of misleading information available on the same website.

In our study, the comparison of the HR and LR groups confirms the validity of the YouTube algorithm in ranking videos during a search inquiry. Interestingly, no significant differences were observed in the total VIQI score between the two groups, probably because current technology enables any person to produce a high quality video. However, the HR group fared better in comparison of the ‘flow’ and ‘precision’ components of the VIQI score. This is likely because flow and appropriate title assignment lead to a higher watch time, which is already a YouTube search result criteria.

Medical information and content index scores were higher in the HR vs. the LR group. Specifically, videos that discuss clinical symptoms in most details have the greatest impact on video inclusion into the HR group, showing that people utilize resources such as YouTube to better understand clinical symptoms of a disease rather than prevalence, outcomes and other attributes of the disease, and suggesting that video producers need to assign more video time on the discussion of clinical symptoms.

The concerning observation that our study brings forth is the fact that amongst the top 100 videos on EHF there were none from academic medical centers or hospitals. Studies on chronic disease information delivery via YouTube have shown that university channels provided about 21 % of the data and were considered sources of useful information (Singh et al. 2012). We feel that government, news agencies, and academic medical centers can provide videos with reliable medical information (and therefore higher MICI scores), which would translate, based on our results, into a higher ‘relevance’ for the viewers. This suggests that our academic medical centers should recognize the YouTube as an important tool for reliable information broadcasting, especially during epidemics. If credible organizations do not initiate accurate and precise information delivery via multimedia channels such as YouTube, independent opportunists may utilize multimedia channels to drive forth their own agendas, taking advantage of the public anxiety that usually accompanies such epidemics.

Like all cross-sectional studies, this study suffers from the limitations of a ‘snapshot’ approach to data collection. YouTube content is dynamic and therefore search inquiry results are ever changing as interests and video watch times perpetually shift over time. However, since we collected data 2 months after the climax of the wide-spread public panic in the U.S. (by which time all video statistics had already plateaued), we were able to minimize the possibility that large panic-driven changes in view times and other variables affected the results. Also, there is a finite possibility that the 100 videos that were studied do not completely represent the available information on EHF.

Between September 2014 and the first week of January 2015, a total of 60,308 people tested positive for Influenza (CDC 2015). However, the focus remained centered around EHF and even though exact figures on the cost of ‘Ebola preparedness’ for individual health systems were not made available publicly, a recent newspaper article revealed that the government requested $154 million for the same, with the goal of achieving at least one “Ebola facility” in each state (2015). Therefore, it can be estimated that each state could spend upwards of $3 million, a large proportion of which may be wasted on “false alarms” caused by misinformed patients who present to the emergency department with a common cold, thinking that they may have contracted EHF. Potential exposures to preventable healthcare associated infections during such unnecessary visits and admissions also impact healthcare expenditure, although further data is needed to assess this negative outcome. On the contrary, the cost of developing informative multimedia to educate communities and prevent such unnecessary visits would probably cost less.

In the era of cost-conscious care, universities and academic centers should consider dissemination of reliable information through the social media and video sharing websites, especially in countries with good penetration of the Internet and social media. Channeling healthcare funds towards such efforts could potentially reduce unnecessary medical visits and, as a consequence, hospital admissions for diseases mistaken to be part of ongoing epidemics.
